# Comprehensive analysis of the MIR4435-2HG/miR-1-3p/MMP9/miR-29-3p/DUXAP8 ceRNA network axis in hepatocellular carcinoma

**DOI:** 10.1007/s12672-021-00436-3

**Published:** 2021-10-07

**Authors:** Li Zhang, Shangshang Hu, Jiasheng Chen, Shasha Ma, Fanghong Liu, Chuanmiao Liu, Yu Gao

**Affiliations:** 1 Department of Infectious Diseases, First Affiliated Hospital of Bengbu Medical College, Bengbu Medical College, 233030 Bengbu, China; 2 National Clinical Research Center for Infectious Diseases, First Affiliated Hospital of Bengbu Medical College, Bengbu Medical College, 233030 Bengbu, China; 3 Research Center of Clinical Laboratory Science, School of Laboratory Medicine, Bengbu Medical College, Bengbu, 233030 China; 4 School of Life Science, Bengbu Medical College, No. 2600, Donghai Road, Bengbu, 233030 China; 5 Anhui Province Key Laboratory of Translational Cancer Research, Bengbu Medical College, Bengbu, 233030 China

**Keywords:** Hepatocellular carcinoma, ceRNA, Prognosis, MIR4435-2HG, MMP9, DUXAP8

## Abstract

A growing number of studies have shown that competitive endogenous RNA (ceRNA) regulatory networks might play important roles during the process of hepatocellular carcinoma (HCC). This study assessed the role of the ceRNA network in immune cell infiltration in HCC. Immune-related gene sets were downloaded from Molecular Signatures Database, and differentially expressed genes were screened based on TCGA HCC transcriptome data. The corresponding miRNAs with low expression and good prognostic implications, and the corresponding lncRNAs with high expression and poor prognostic were identified to construct ceRNA networks. The networks were utilized for clinical correlation analysis and risk model construction, and the CIBERSORT algorithm was applied to assess immune cell infiltration. In this study, the mRNA-miRNA-lncRNA model was used to construct a ceRNA network in HCC using immune-related differentially expressed mRNAs. Assessment of the MIR4435-2HG/hsa-miR-1-3p/MMP9/hsa-miR-29-3p/DUXAP8 ceRNA network axis in HCC showed that a high risk/poor prognosis was significantly correlated with tumor stage and invasion depth. MMP9 was positively correlated with resting M0 macrophages and NK cells and negatively correlated with activated mast cells, resting mast cells, monocytes and activated NK cells. DUXAP8 was positively correlated with M2 macrophages and negatively correlated with MIR4435-2HG, which was positively correlated with M2 macrophages and negatively correlated with activated mast cells, CD8 T cells and follicular helper T cells. The correlation of the MIR4435-2HG/hsa-miR-1-3p/MMP9/hsa-miR-29-3p/DUXAP8 ceRNA network axis with immune cell infiltration provides further information on the mechanism of HCC development. The result might improve our understanding the interactions between immune related genes and non-coding RNAs in the occurrence and development of HCC, and the relevant RNAs might be used as diagnostic and prognostic biomarkers and molecular targets in HCC patients.

**Supplementary Information:**

The online version contains supplementary material available at 10.1007/s12672-021-00436-3.

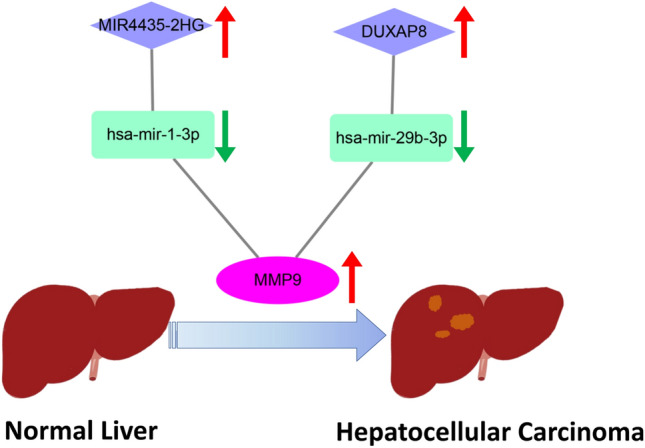

## Introduction

Primary liver cancer is one of the most common malignancies, and approximately 80% of primary liver cancers are hepatocellular carcinoma (HCC) [1]. There are many treatment options for liver cancer, including surgical resection, drug therapy, liver transplantation, and early treatment [2]. However, most liver cancers are already in an advanced stage at diagnosis, so the prognosis is extremely poor; surveys show that the 2-year survival rate of liver cancer patients is less than 50%, while the 5-year survival rate is approximately 10% [3]. With in-depth research on the mechanism of liver cancer, there has been a great development in the treatment of liver cancer, and now the role of tumor immune cells in liver cancer has been well characterized. In the tumor microenvironment, immune cells can recognize tumor cells and can remove them or promote their development [4]. Tumor immunotherapy is the use of immune principles to activate or strengthen the body’s immune system to remove or inhibit the development of cancer cells in the body [5]. Tumor immunotherapy currently holds good promise for many cancers and has been extensively studied in liver cancer [6]. Immune checkpoint inhibitors, such as anti-PD-1, anti-PD-L1, and anti-CTLA-4 antibodies, have been used in the clinic and have shown promising efficacy in patients with advanced liver cancer [7]. Therefore, exploring the mechanisms related to immune infiltration can facilitate the development of liver cancer treatment.

Recently, accruing results suggested that long non-coding RNAs (lncRNAs) and microRNAs (miRNAs) could play essential roles in the proliferation, migration, and invasion of various tumor cells [8]. And the interactions of mRNA, miRNA, and lncRNA play multiple roles in the development of liver cancer [9]. The competing endogenous RNA (ceRNA) network has a major regulatory role in liver cancer, and lncRNAs act as endogenous molecular sponges competing for the binding of miRNAs and regulating the expression levels of mRNAs, which in turn affects the development of HCC [10]. For example, mCM3AP-AS1 in HCC targets miR-194-5p to promote the expression of FOXA1 and exert oncogenic effects [11]. Another study also constructed a ceRNA network related to HCC recurrence, and revealed that four mRNAs (ADH4, DNASE1L3, HGFAC and MELK) could be used as potential biomarkers for HCC recurrence prediction and targeted therapies [12]. However, the immune cell-related mRNA-miRNA-lncRNA ceRNA network in HCC has not been reported. The aim of this study was to identify an immune related competing endogenous RNA (ceRNA) regulatory axis in HCC through comprehensive bioinformatics analysis. The finding of this study can be helpful in improving our understanding of the roles and underlying mechanisms of the interactions between immune related genes and non-coding RNAs in the occurrence and development of HCC, and the relevant RNAs may be used as diagnostic and prognostic biomarkers and molecular targets in HCC patients.

## Materials and methods

### Analysis process of this study

This study was carried out by using comprehensive bioinformatics analysis. Firstly, immune-related differentially expressed mRNAs (immune-DEmRNAs) were identified through immune gene-related sets from Molecular Signatures Database (MSigDB) v7.4 [13] and the expression data of hepatocellular carcinoma from The Cancer and Tumor Genome Atlas (TCGA) database (https://www.cancer.gov/tcga). Then, gene function enrichment and survival analyses of immune-DEmRNAs in HCC were performed. Considering the expression and survival analyses of immune-DEmRNAs, the key genes were selected for subsequent study. The upstream miRNAs and lncRNAs were predicted by using online bioinformatic tools, and the immune cell-related mRNA-miRNA-lncRNA ceRNA network was constructed. Subsequently, the constructed ceRNA networks were analyzed for clinical correlation assessment and risk model construction, and the CIBERSORT algorithm was used to analyze the proportion of infiltrating immune cells in TCGA tumor samples. Finally, the relationships between the genes in the ceRNA network and the prognosis and infiltration of immune cells in HCC were assessed.

### Data acquisition

Immune gene-related sets (IMMUNE_RESPONSE.gmt and IMMUNE_SYSTEM_PROCESS.gmt) (version 5.2) were acquired from Molecular Signatures Database v7.4 on Gene Set Enrichment Analysis (GSEA) website (http://www.gsea-msigdb.org/gsea/index.jsp) [14]. The expression data of mRNA and lncRNA (tumor: n  =  374, normal: n  =  50) and miRNA (tumor: n  =  374, normal: n  =  50) were downloaded from The Cancer Genome Atlas (TCGA database, https://portal.gdc.cancer.gov, Version 29.0-February 2, 2021).The relevant clinical data, including, age, sex, grade, TNM stage, invasion depth (T), lymph node metastasis (N) and distant metastasis (M) (n  =  235), and survival time (n  =  370), were also obtained from TCGA database The patients involved in the database have obtained ethical approval. Users can download relevant data for free for research and publish relevant articles. This study is based on open-source data, so there are no ethical issues and other conflicts of interest.

### Screening of differentially expressed mRNAs, lncRNAs, and miRNAs

The online tool of Gene Expression Profiling Interactive Analysis (GEPIA) [15] was used to identify the differentially expressed mRNAs, lncRNAs and miRNAs by combined the relevant data from TCGA database and Genotype-Tissue Expression (GTEx) database (version 8) (https://gtexportal.org) [16], with |log_2_-Fold Change (FC)| >  1 and adjust P value  <  0.05. The immune-related differentially expressed mRNAs (immune-DEmRNAs) were identified by compared the immune gene-related sets (IMMUNE_RESPONSE.gmt and IMMUNE_SYSTEM_PROCESS.gmt) with the list of differentially expressed mRNAs in HCC.

### Gene ontology (GO) and Kyoto Encyclopedia of Genes and Genomes (KEGG) analysis

Gene Ontology (GO) is a comprehensive database describing Gene functions, divided into Biological Process, Cellular Component, and Molecular Function. KEGG (Kyoto Encyclopedia of Genes and Genomes) is a comprehensive database incorporating genome, chemistry, and system function information. To reveal the function of immune-DEmRNAs, GO and KEGG analyses were performed using the packages of clusterProfiler, org.Hs.eg.db, enrichplot, and ggplot2 with R software (version 4.0.2). The enrichment was considered to be significant when the corrected p-value was less than 0.05.

### Protein–protein interaction (PPI) network construction

STRING is a database for the analysis of functional protein association networks [17]. The PPI network for immune-DEmRNAs was constructed using STRING v11.5 (https://www.string-db.org/). The PPI network was set with a minimum required interaction score of 0.4, and unconnected nodes were removed. The STRING protein network files were visualized using Cytoscape software (version 3.8.2) [18].

### ceRNA network construction

The mRNAs with significant differential expression levels and prognostic roles were screened in the PPI network of up- and down-regulated immune-DEmRNAs. miRNAs upstream of mRNAs were predicted using the miRNet 2.0 online tool (https://www.mirnet.ca/), which is a miRNA-centric network visual analytics platform [19]. miRNAs with significant differential expression and prognostic role were screened. The miRNet online tool was used to predict eligible miRNAs for lncRNAs and subsequently screened for prognostically useful lncRNAs (P  <  0.05) based on TCGA liver cancer samples using one-way Cox regression analysis. Finally, the eligible mRNAs, miRNAs, and lncRNAs were subjected to ceRNA network construction, followed by extraction of subnetwork axes containing significantly differentially expressed lncRNAs.

### Survival analysis

The overall survival (OS) of the differentially expressed mRNA or lncRNA was analyzed by using the survminer and survival R packages, based on TCGA data for HCC (excluding patient information with no survival time). The patients were split into two groups (high-expressed group and low-expressed group) by using median expression as the cut-off value. The overall survival curve analysis of the differentially expressed miRNA was performed by using the online tool of Kaplan–Meier plotter [20]. On the main interface of the Kaplan–Meier plotter, “Start miRpower for liver cancer” in “miRNA” was selected; the miRNA name to be searched was inputted and the default values for other options were selected. The patients were split into two groups by using the “Auto select best cutoff” option. The log-rank P value and hazard ratio (HR) were calculated.

### Clinical correlation analysis and risk model construction

Based on TCGA liver cancer clinical samples, the R software limma package and ggpubr package were used for clinical characterization of the main genes of the ceRNA network axis, including age, sex, tumor stage, tumor infiltration, lymphatic metastasis and distant metastasis. Multivariate Cox analysis was performed on the main genes of the ceRNA network axis using the survival R package, and the genes with a P value  <  0.05 were selected to construct a risk model, where risk score  =  survival-related lncRNA1 coefficient × lncRNA1 expression  +  lncRNA2 coefficient × lncRNA2 expression  + ….. +  lncRNAn coefficients × lncRNAn expression. Survival analysis was performed on the risk model, and the R software package timeROC was used to plot ROC curves to assess the accuracy of the prognosis of this estimated risk model. Univariate and multivariate Cox regression analyses were performed to assess clinical characteristics based on the risk model. The Wilcoxon rank-sum or Kruskal–Wallis rank-sum test was used as the statistical significance test with a P value  <  0.05 indicating statistical significance.

### Immune cell infiltration analysis

The percentage of tumor-infiltrating immune cells in all HCC samples from the TCGA database were calculated using the cell type identification by estimating relative subsets of RNA transcripts (CIBERSORT) algorithm [21]. The multiple comparisons error adjustment was performed by using the Benjamini–Hochberg false discovery rate (FDR) approach, and then samples were screened according to adjust P  <  0.05. In this study, 56 HCC samples were selected at the cutoff P value, and that this P value represented the statistical significance of the deconvolution of particular sample by CIBERSORT. Then, a correlation analysis between gene expression and tumor immune cells in the 56 HCC samples was conducted by using R software packages ggplot2, ggpubr, and ggExtra, and the Pearson coefficient was used for significance testing.

### Statistical analysis

Statistical analysis was performed using R software (version 4.0.2). Independent sample t test was employed to analyze the differential expression levels of mRNAs, lncRNAs and miRNAs in HCC patients and control individuals. The overall survival (OS) of the differentially expressed mRNA or lncRNA was analyzed by using the survminer and survival R packages. The overall survival of miRNA was performed using Kaplan–Meier plotter. The Wilcoxon rank-sum test was applied for the analysis of the difference between tumor immune cells and gene expression level in HCC. A value of P  <  0.05 was considered statistically significant.

## Results

### Screening of immune-related, differentially expressed mRNAs (immune-DEmRNAs) for hepatocellular carcinoma

Immune-related gene sets were obtained from Molecular Signatures Database v7.4 on Gene Set Enrichment Analysis (GSEA) website. There were 235 genes in IMMUNE_RESPONSE gene set and 332 genes in IMMUNE_SYSTEM_PROCESS gene set. According to TCGA HCC data, 7168 differentially expressed mRNAs were identified. Among them, 45 immune-related, differentially expressed mRNAs (immune-DEmRNAs) were found in both datasets. Compared with normal liver tissue samples, 28 immune-DEmRNAs were upregulated and 17 immune-DEmRNAs were downregulated in HCC tissue samples (Supplementary Table 1).

### Gene ontology (GO) and KEGG pathway analyses of the immune-DEmRNAs

**Fig. 1:**
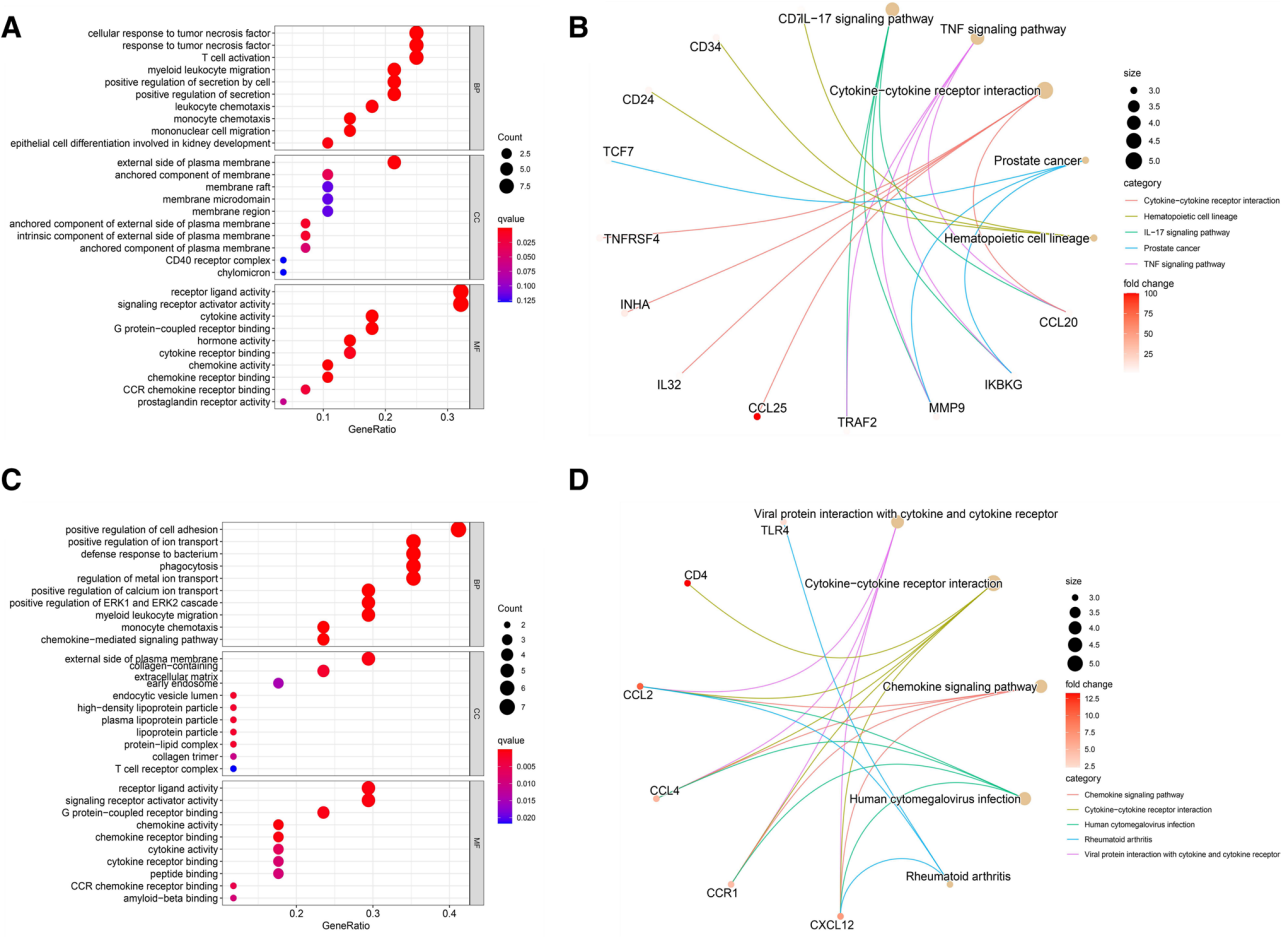
Gene ontology (GO) and Kyoto Encyclopedia of Genes and Genomes (KEGG) analysis of immune-related up- and down-regulated genes in HCC. **a**, **b** showed GO and KEGG analyses of upregulated genes in HCC, respectively. **c**, **d** showed GO and KEGG analyses of downregulated genes in HCC, respectively

To further analyze the biological functions and related pathways of immune-DEmRNAs, GO and KEGG analyses were performed. As shown in Fig. 1a, the upregulated gene-related GO terms were the cellular response to tumor necrosis factor, the external side of the plasma membrane, receptor-like activity, and so on. As shown in Fig. 1b, the upregulated genes associated with KEGG pathways included cytokine-cytokine receptor interaction, hematopoietic cell lineage, IL-17 signaling pathway, and TNF signaling pathway. As shown in Fig. 1c, the downregulated gene-related GO terms were the positive regulation of cell adhesion, the external side of plasma membrane, chemokine activity, and so on. As shown in Fig. 1d, the downregulated gene-related KEGG pathways included the chemokine signaling pathway, cytokine-cytokine receptor interaction, human cytomegalovirus infection, viral protein interaction with cytokine and cytokine receptor.

### PPI network construction and further analysis of immune-DEmRNAs

**Fig. 2:**
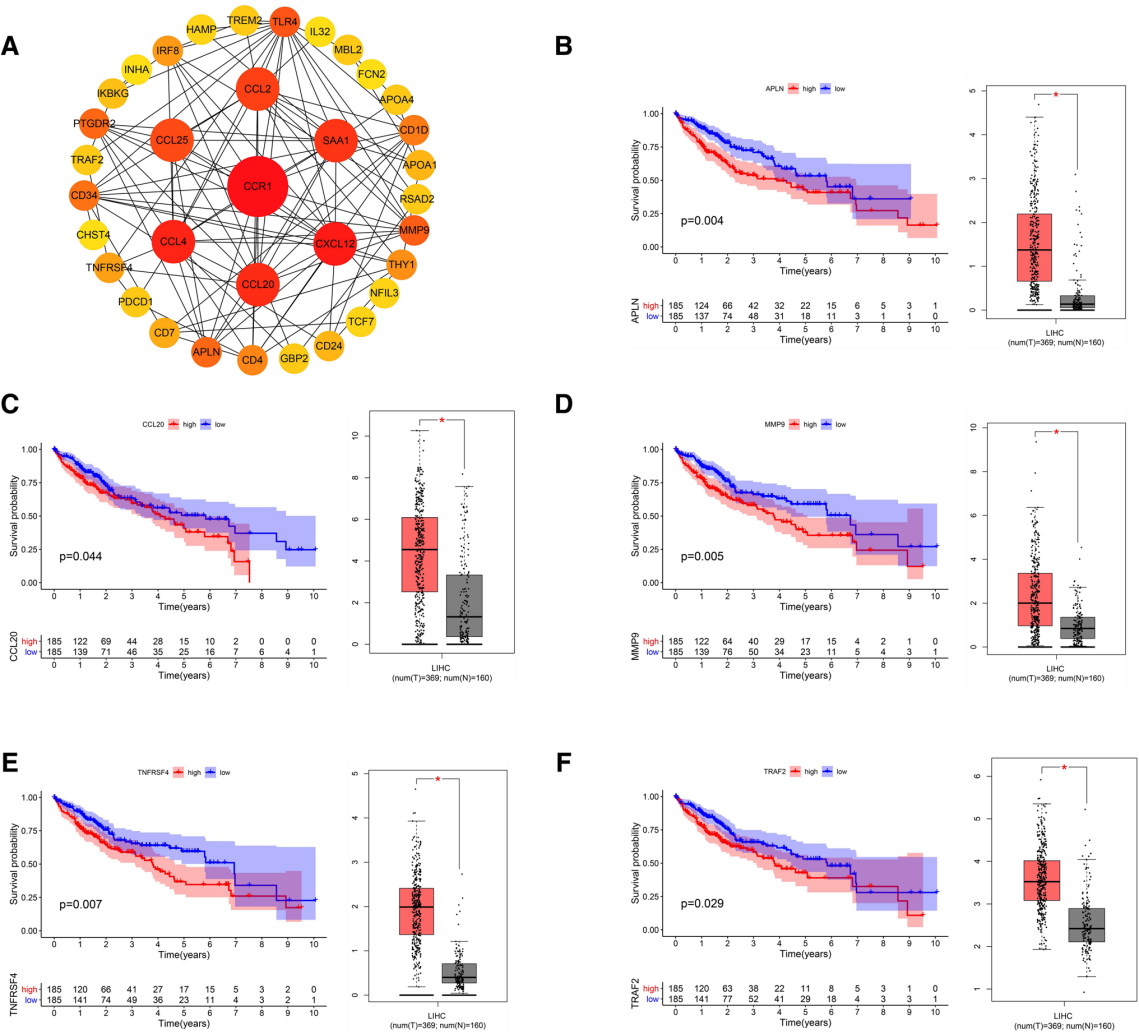
mRNAs with significant differential expression and prognostic implications in HCC. **a** Protein network diagram of Immune-DEmRNAs. Differential expression and survival analysis of APLN (**b**), CCL20 (**c**), MMP9 (**d**), TNFRSF4 (**e**), and TRAF2 (**f**) in HCC (*P  <  0.01)

As shown in Fig. 2a, the immune-DEmRNA PPI network was constructed based on the Search Tool for the Retrieval of Interacting Genes/Proteins (STRING) database. The PPI network included 19 upregulated genes (CCL20, CD34, APLN, CCL25, CD7, PTGDR2, THY1, TNFRSF4, CD24, IKBKG, MMP9, TRAF2, INHA, PDCD1, TCF7, APOA4, IL32, TREM2, and GBP2), and 16 downregulated genes (TLR4, CCL2 CCR1, CCL4, SAA1, CXCL12, CD4, IRF8, CD1D, APOA1, MBL2, HAMP, RSAD2, FCN2, NFIL3, and CHST4). These genes were further analyzed using the Gene Expression Profiling Interactive Analysis (GEPIA) differential expression module and the TCGA dataset containing information on survival time for liver cancer patients (n  =  370). Among the 35 upregulated and downregulated genes, five genes (APLN, CCL20, MMP9, TNFRSF4, and TRAF2) were highly expressed in HCC and predicted a poor prognosis (Fig. 2b–f).

### Prediction and screening of miRNAs upstream of mRNAs

**Fig. 3:**
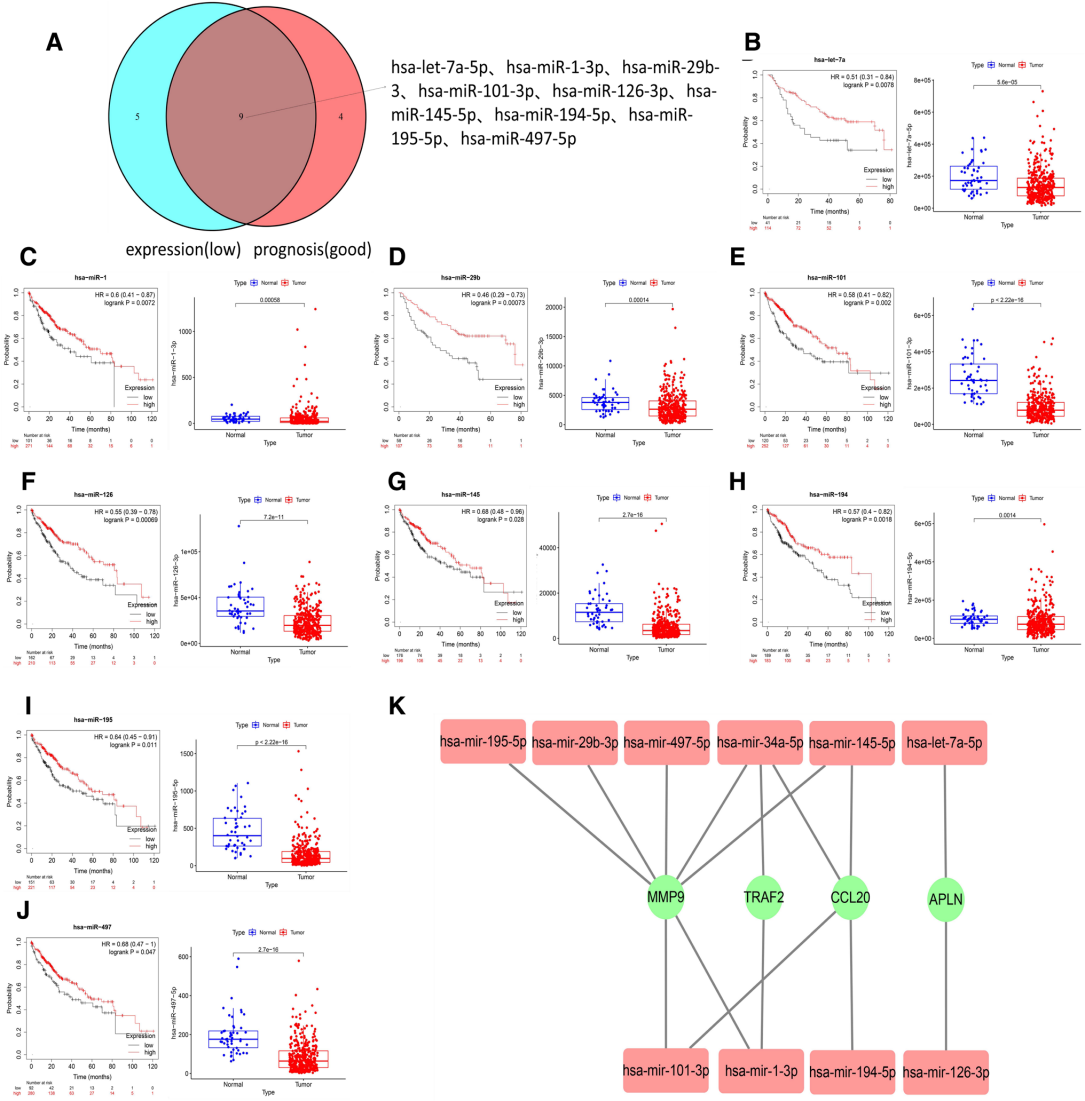
miRNAs with significant differential expression and prognostic implications in HCC. **a** Nine miRNAs that were expressed at low levels in HCC and predict good prognosis in HCC patients were integrated by Venn diagram. The differential expression and prognostic implications of these nine miRNAs hsa-mir-29b-3p (**b**), hsa-mir-145-5p (**c**), hsa-mir-195-5p (**d**), hsa-mir-497-5p (**e**), hsa-mir-1-3p (**f**), hsa-mir-101-3p (**g**), hsa-let-7a-5p (**h**), hsa-mir-126-3p (**i**), and hsa-mir-194-5p (**j**) were analyzed in HCC. **k** The interaction between mRNA and miRNA

To predict miRNAs upstream of 5 genes (APLN, CCL20, MMP9, TNFRSF4, and TRAF2), the miRNet online tool was used. The total of 25 upstream miRNAs were identified (Supplementary Table S2). According to the previous section result of that mRNAs were highly expressed in HCC and predicted a poor prognosis in HCC patients, miRNAs should theoretically be expressed at low levels in HCC and predict a good prognosis in HCC patients. Among 25 miRNAs, the expression levels of 14 miRNAs were significantly lower in HCC patients, and 10 miRNAs were predicted a good prognosis by using Kaplan–Meier plotter online tools. The hazard ratio for death and p value were listed in Supplementary Table S3. The intersection of both was taken; nine candidate miRNAs (hsa-mir-29b-3p, hsa-mir-145-5p, hsa-mir-195-5p, hsa-mir-497-5p, hsa-mir- 1-3p, hsa-mir-101-3p, hsa-let-7a-5p, hsa-mir-126-3p, and hsa-mir-194-5p) were screened out and a volcano plot was shown in Fig. 3a. The differential expression and prognostic implications of these nine miRNAs were shown in Fig. 3b–j. A network diagram was constructed for these nine miRNAs and the corresponding mRNAs (Fig. 3k). No eligible upstream miRNA of TNFRSF4 was identified.

### Prediction and screening of lncRNAs upstream of miRNAs and ceRNA network construction

**Fig. 4:**
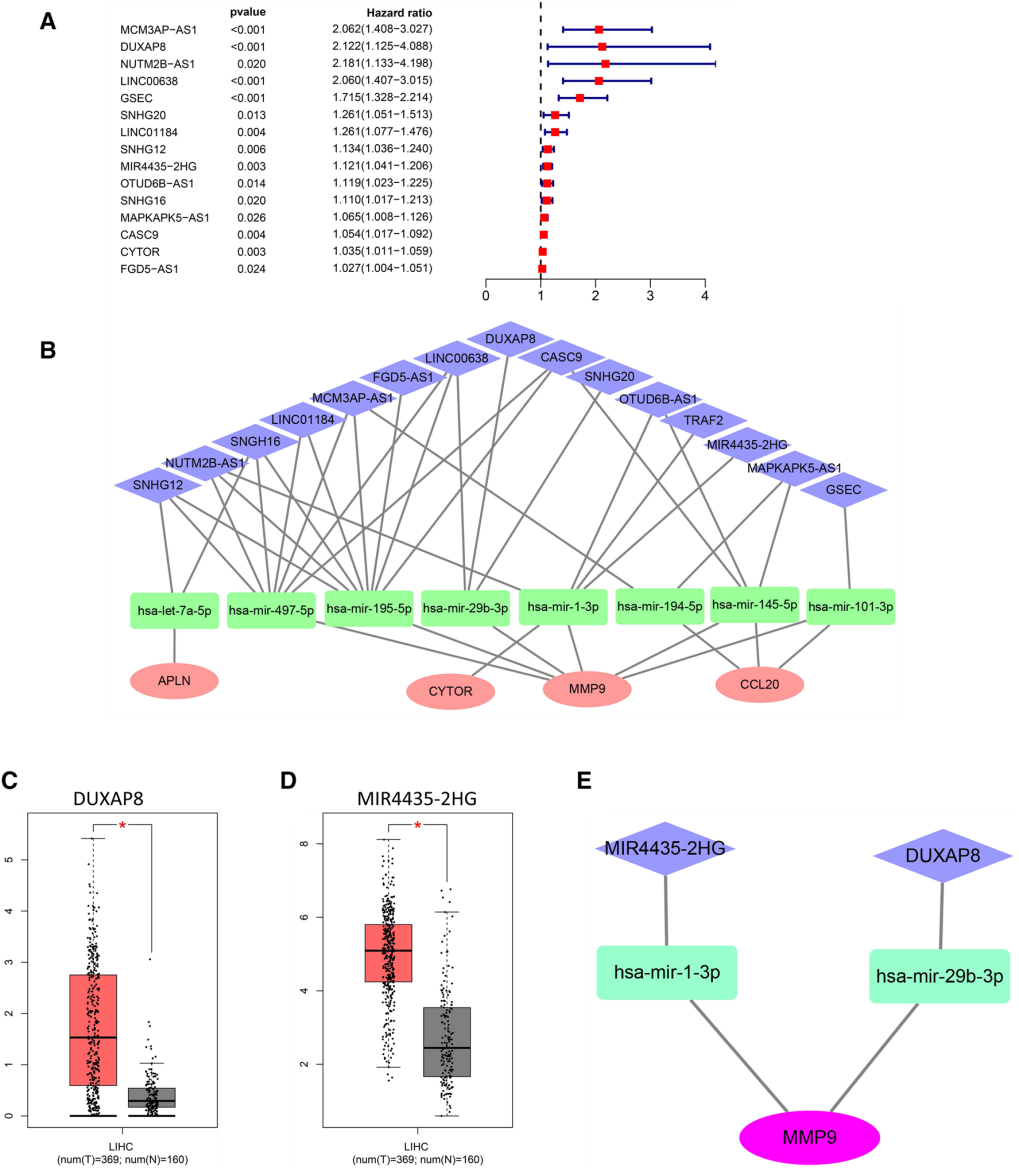
Identification of upstream lncRNAs of miRNAs and the construction of ceRNA network. **a** Screening of 15 lncRNAs related to a poor prognosis in liver cancer patients. **b** Construction of corresponding ceRNA networks based on these 15 lncRNAs. DUXAP8 (**c**) and MIR4435-2HG (**d**) were highly expressed in HCC. **e** The ceRNA MIR4435-2HG/hsa-miR-1-3p/MMP9/hsa-miR-29-3p/DUXAP8 network axis

The upstream lncRNAs of 9 miRNAs (hsa-mir-29b-3p, hsa-mir-145-5p, hsa-mir-195-5p, hsa-mir-497-5p, hsa-mir-1-3p, hsa-mir-101-3p, hsa-let-7a-5p, hsa-mir-126-3p, and hsa-mir-194-5p) were predicted by using the miRNet online tool. A total of 289 lncRNAs were predicted based on these 9 miRNAs, and subsequent one-way Cox regression analysis of these 289 lncRNAs showed that 15 lncRNAs were related to a poor prognosis in liver cancer patients (Fig. 4a). The corresponding ceRNA network was constructed based on these 15 lncRNAs (Fig. 4b), but there were no eligible lncRNAs for hsa-mir-126-3p. Theoretically, upstream lncRNAs should have a poor prognostic implication and significantly higher expression in HCC patients than in control individuals. In this study, the differential expression levels of the 15 lncRNAs in HCC were assessed, and two lncRNAs (DUXAP8 and MIR4435-2HG) were identified with higher expression levels in HCC (Fig. 4c, d). According to these results, a ceRNA network axis, the MIR4435-2HG/hsa-miR-1-3p/MMP9/hsa-miR-29-3p/DUXAP8 axis, was constructed, and it was used for further analysis in the next step (Fig. 4e).

### Construction of a risk model for the MIR4435-2HG/hsa-miR-1-3p/MMP9/hsa-miR-29-3p/DUXAP8 ceRNA network axis

**Fig. 5:**
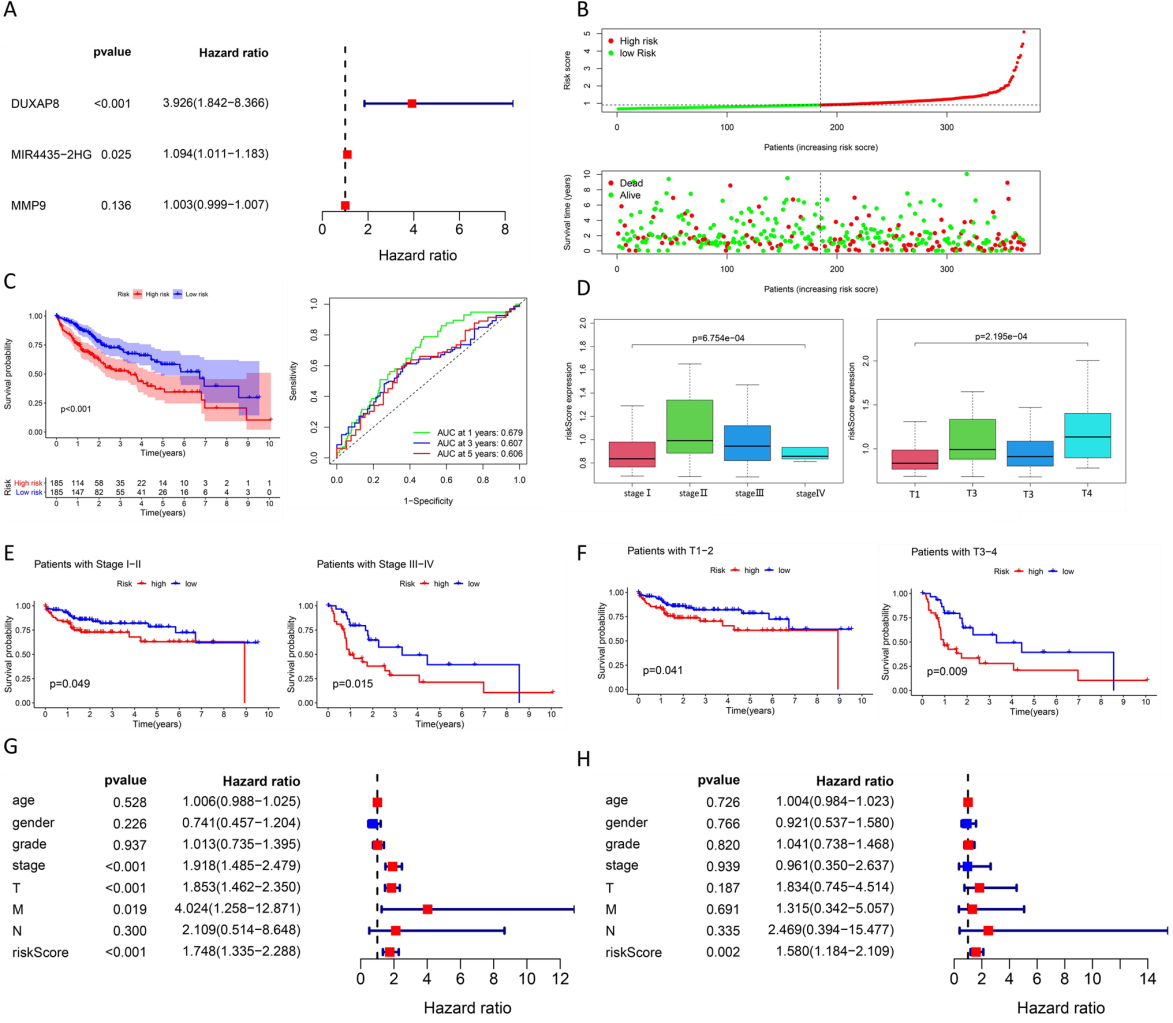
Construction of risk models for MIR4435-2HG and DUXAP8. **a** Multifactorial Cox regression analysis of MIR4435-2HG, MMP9, and DUXAP8. **b** Classification of HCC patients into two groups (high-risk and low-risk groups) according to the median risk scores. **c** Classification of liver cancer patients into high-risk and low-risk groups according to the median risk scores. **d** Correlation of TNM stage and depth of invasion (T) with risk scores. **e** Survival curves for the stage I–II (n  =  163) and stage III–IV (n  =  72) groups. **f** Survival curves for the T1–2 (n  =  167) and T3–4 (n  =  68) groups. **g** Univariate Cox and **h** multivariate Cox regression analysis

Multivariate Cox regression analysis of the expression levels of MIR4435-2HG, MMP9 and DUXAP8 showed that MIR4435-2HG and DUXAP8 were reliable prognostic indicators in HCC patients (P  <  0.05) (Fig. 5a).Based on the correlation coefficient between the expression of these two genes, a risk model was constructed: risk score  =  (1.37 × expression value of DUXAP8 +  0.0896 × expression value of MIR4435-2HG) (Fig. 5b). Subsequently, HCC patients were divided into high-risk and low-risk groups according to the median risk score, and then survival analysis was performed for these two groups. As shown in Fig. 5c, the survival of the high-risk group was significantly smaller than that of the low-risk group (P  <  0.01). Based on the risk model, the correlation between the risk scores and clinical characteristics was analyzed, and the result showed that the risk scores were significantly correlated with TNM stage and invasion depth (T) (P  <  0.05) (Fig. 5d). The patients were then divided into two different subgroups, TNM stage I-II and stage III–IV, for survival analysis; as shown in Fig. 5e, the survival rates of the high-risk groups in both the stage I–II and stage III–IV subgroups were significantly less than that of the low-risk groups (P  <  0.05). The patients were then divided into two different subgroups, T1–2 and T3–4, and the survival rates of the high-risk groups in both the T1–2 and T3–4 subgroups was significantly lower than that of the low-risk groups (P  <  0.05) (Fig. 5f). Therefore, TNM stage and invasion depth were considered as potential prognostic factors for the risk model. Then, candidate variables for the risk model were assessed by performing univariate Cox regression and multivariate Cox regression analysis of the clinical characteristics. In the univariate Cox regression analysis, TNM stage, invasion depth, metastatic distance, and risk score were prognostic factors for overall survival in HCC in this risk model (P  <  0.05) (Fig. 5g). In the multivariate Cox regression analysis, only the risk score was a prognostic factor for this risk model (P  <  0.05) (Fig. 5h). Taken together, the results suggested that the abnormal expression of MIR4435-2HG and DUXAP8 might be closely associated with the development of HCC. As shown in the graphical abstract, MIR4435-2HG and DUXAP8 were highly expressed in HCC tissues, and survival time was significantly lower in the high expression group. It suggested that MIR4435-2HG might compete for the binding of has-miR-1-3p, thereby promoting the expression of MMP9 to induce oncogenic effects. Moreover, DUXAP8 also might promote MMP9 expression by competing for binding has-mir-29b-3p.

### Analysis of the correlations between the MIR4435-2HG/hsa-miR-1-3p/MMP9/hsa-miR-29-3p/DUXAP8 ceRNA network axis and tumor immune cell infiltration in HCC

**Fig. 6:**
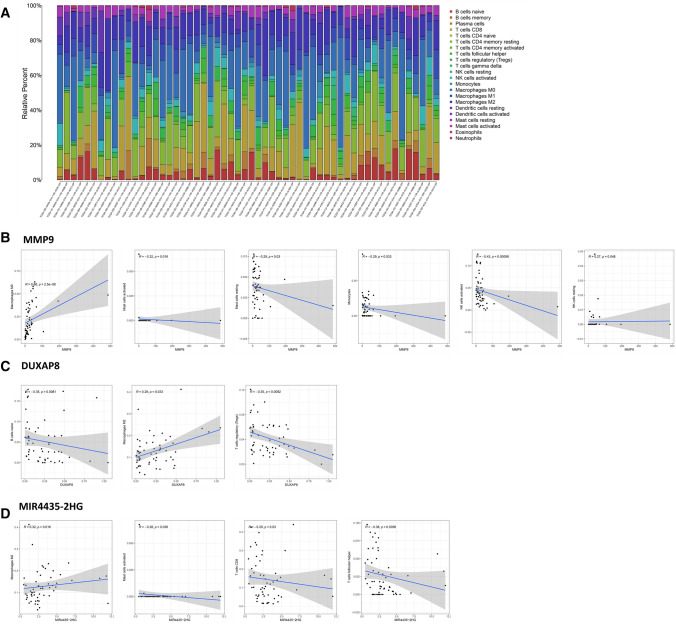
Correlation of the ceRNA network axis with tumor immune cells. **a** Proportion of 22 tumor immune cells in TCGA liver cancer samples (n  =  56). Scatter plot showing the correlation of MMP9 (**b**), DUXAP8 (**c**), and MIR4435-2HG (**d**) with tumor immune cells

To explore the correlation of the expression levels of MIR4435-2HG, MMP9 and DUXAP8 with tumor-infiltrating immune cells, the proportions of immune cell subsets were identified using the CIBERSORT algorithm, and 22 immune cells in HCC samples and the correlation between them were analyzed. The distribution of tumor-infiltrating immune cell (TIC) abundance in all HCC samples was estimated by CIBERSORT algorithm, and 56 HCC samples with adjust P  <  0.05 were selected for the following analysis (Fig. 6a). Correlation analysis showed that MMP9 was positively correlated with resting M0 macrophages and NK cells and negatively correlated with activated mast cells, resting mast cells, monocytes and activated NK cells (Fig. 6b). DUXAP8 was positively correlated with M2 macrophages and negatively correlated with naive B cells and regulatory T cells (Fig. 6c). MIR4435-2HG was positively correlated with M2 macrophages and negatively correlated with activated mast cells, CD8 T cells and follicular helper T cells (Fig. 6d).

## Discussion

In recent decades, significant progress has been made in the early diagnosis, surgical treatment, and liver transplantation for HCC, and the incidence and mortality of HCC have been significantly reduced; however, the prognosis of HCC remains poor, the mortality rate is still high, and the mechanism of HCC development is still unclear [22]. It is crucial to develop efficient treatment methods and to elucidate the molecular mechanisms of HCC. Immunotherapy has great potential in the treatment of tumors, and great progress has been made in the treatment of HCC [23]. A clear understanding of the link between tumor immune cells and tumor molecular mechanisms could facilitate improvements in the effectiveness of immunotherapy. The ceRNA network hypothesis suggests that lncRNAs act as endogenous molecular sponges competing for the binding of miRNAs and regulating the expression levels of mRNAs, which in turn affects tumor development [9]. For example, HOXD-AS1 was found to competitively bind miR-130a-3p and inhibit the degradation of the target gene SOX4, thus promoting the metastasis of HCC cells [24]. However, ceRNA networks are still understudied in HCC. In this study, an immune related competing endogenous RNA (ceRNA) regulatory axis (MIR4435-2HG/hsa-miR-1-3p/MMP9/hsa-miR-29-3p/DUXAP8) in HCC was constructed through comprehensive bioinformatics analysis. In this ceRNA network axis, each RNA was significantly differentially expressed and had a prognostic role in liver cancer. Two miRNAs (hsa-miR-1-3p and hsa-miR-29-3p) were expressed at low levels in liver cancer and were related to a good prognosis for liver cancer patients. MMP9 and two lncRNAs (MIR4435-2HG and DUXAP8) were highly expressed in HCC and were associated with a poor prognosis in patients with HCC and with immune tumor cell infiltration.

In the present study, the mRNA-miRNA-lncRNA model was used for the first time to construct a ceRNA network in HCC using immune-related mRNAs. Among the MIR4435-2HG/hsa-miR-1-3p/MMP9/hsa-miR-29-3p/DUXAP8 ceRNA network axis components, MMP9 is a member of the matrix metalloproteinase (MMP) family, a class of metal ion-dependent proteases that increases the invasive and migratory ability of tumor cells because MMPs degrade a variety of proteins in the cell periphery [25]. In this study, MMP9 was highly expressed in HCC and was related to a poor prognosis for patients with HCC. Moreover, the expression level of MMP9 was significantly correlated with sex, grade, TNM stage, and invasion depth in HCC. The previous report showed that high expression of MMP9 could promote metastasis of tumor cells, promotes tumor angiogenesis, and inhibits apoptosis [26]. LncRNA FLJ33360 has been reported to inhibit miRNA-140 to increase the expression of MMP9 to accelerate the development of HCC [27]. In this study, the two miRNAs (hsa-miR-1-3p and hsa-miR-29-3p) were identified with lower expression levels in HCC and they were related to a good prognosis for HCC patients. The results of the study on hepatoma cell lines showed that overexpression of hsa-miR-1-3p could promote the apoptosis and inhibit the proliferation of hepatoma cells. Further analysis showed that hsa-miR-1-3p could inhibit Sox9 to achieve an antitumor effect [28]. However, there is no study on the role of hsa-miR-29-3p in HCC. Another study showed that hsa-miR-29-3p was expressed at low levels in nasopharyngeal carcinoma and could target COL1A1 to improve the radiosensitivity of nasopharyngeal carcinoma cells [29].

In this study, according to the MIR4435-2HG/hsa-miR-1-3p/MMP9/hsa-miR-29-3p/DUXAP8 ceRNA network axis, a risk model was constructed for MIR4435-2HG and DUXAP8, and the survival rate of the high-risk patient group was significantly lower than that of the low-risk group. Moreover, TNM stage and invasion depth were potential prognostic factors in the risk model, and the risk score could be used as an independent prognostic indicator for patients with HCC. In a recent study, MIR4435-2HG was found to be highly expressed in HCC tissues; survival time was significantly lower in the high expression group, and MIR4435-2HG was found to compete for the binding of miR-22-3p, thereby promoting the expression of YWHAZ to induce oncogenic effects [30]. DUXAP8 binding to miR-490-5p accelerated the expression of the oncogene BUB1, further promoting the value added and migration of HCC cells [31]. In terms of the diagnostic value in liver cancer, the expression of MMP9, MIR4435-2HG, and DUXAP8 has the potential to differentiate between normal individuals and HCC patients. Taken together, the MIR4435-2HG/hsa-miR-1-3p/MMP9/hsa-miR-29-3p/DUXAP8 ceRNA network axis might be closely associated with the development of HCC.

In the correlation analysis of tumor immune cell infiltration, MMP9 was positively correlated with M0 macrophages and resting NK cells and negatively correlated with activated mast cells, resting mast cells, monocytes and activated NK cells. Cox multivariate regression analysis showed that M0 macrophages could be a poor prognostic factor in patients with HCC [32]. The proportions of resting mast cells and activated NK cells were significantly lower in the group with HCC than in the group without normal tissue [33]. In the present study, both DUXAP8 and MIR4435-2HG were positively correlated with M2 macrophages, which have a role in promoting tumor development, angiogenesis, and tumor cell metastasis and help tumor cells evade immune recognition [34, 35]. Several studies have shown that M2 macrophages could play a significant role in the development of HCC. The lncRNA of LINC00662 activated Wnt/β-linked protein signaling in macrophages in a paracrine manner and further promoted M2 macrophage polarization, which further promoted the value added and metastasis of HCC cells and inhibited their apoptosis [36]. Furthermore, the study on HCC cells cultured in M2 macrophage-conditioned medium showed fibroblast-like morphology, increased metastatic capacity and expression of epithelial-mesenchymal transition (EMT) markers and the oncogene TLR4, further promoting HCC cell migration [37]. Therefore, the result of this study suggested that the MIR4435-2HG/hsa-miR-1-3p/MMP9/hsa-miR-29-3p/DUXAP8 ceRNA network axis could be a potential target for immunotherapy in HCC.

In summary, the MIR4435-2HG/hsa-miR-1-3p/MMP9/hsa-miR-29-3p/DUXAP8 ceRNA network axis was constructed by using bioinformatical tools, and it might be closely associated with the development of HCC, associated with a poor prognosis in HCC patients, and associated with tumor immune cell infiltration. The result might offer some insight into the epigenetic processes occurring in high-risk HCC patients. And the relevant RNAs might be used as diagnostic and prognostic biomarkers and molecular targets in HCC patients.

## Supplementary Information

Additional file 1: Supplementary Table 1: The list of immune-related, differentially expressed mRNAs (immune-DEmRNAs) for hepatocellular carcinoma (DOCX 21 KB)

Additional file 2: Supplementary Table 2: The list of upstream miRNAs of five immune-DEmRNAs for hepatocellular carcinoma (DOCX 20 KB)

Additional file 3: Supplementary Table 3: The hazard ratio for death and p value of upstream miRNAs in HCC (DOCX 19 KB)

## Data Availability

Publicly available datasets were analyzed in this study, these can be found in database of The Cancer Genome Atlas (https://portal.gdc.cancer.gov). The authors confirm that the data supporting the findings of this study are available within the article.
