# Supplementary material for: Comprehensive analysis of the MIR4435-2HG/miR-1-3p/MMP9/miR-29-3p/DUXAP8 ceRNA network axis in hepatocellular carcinoma
Source: Discov Oncol. 2021 Oct 7;12:38. doi: 10.1007/s12672-021-00436-3 (PMC8777520; doi:10.1007/s12672-021-00436-3)
Supplement: Supplementary file 1 — Additional file 1: Supplementary Table 1: The list of immune-related, differentially expressed mRNAs (immune-DEmRNAs) for hepatocellular carcinoma (DOCX 21 KB) [file 12672_2021_436_MOESM1_ESM.docx]

**Supplementary Table 1**

The list of immune-related, differentially expressed mRNAs (immune-DEmRNAs) for hepatocellular carcinoma

| Gene ID | logFC | logCPM | PValue | FDR |
| --- | --- | --- | --- | --- |
| FCN2 | -4.619611006 | 8.787947227 | 5.94E-83 | 1.24E-80 |
| CXCL12 | -2.451326287 | 9.095252361 | 3.35E-52 | 3.48E-50 |
| CD4 | -1.744221276 | 9.273061228 | 1.21E-46 | 8.42E-45 |
| THY1 | 2.666304319 | 8.756850819 | 3.58E-36 | 1.86E-34 |
| CD34 | 2.20956989 | 8.144231916 | 1.37E-31 | 5.70E-30 |
| HAMP | -3.47362632 | 10.80925397 | 4.18E-27 | 1.45E-25 |
| IRF8 | -1.269260678 | 7.881830483 | 3.12E-23 | 9.26E-22 |
| APLN | 3.472754412 | 7.65493909 | 1.42E-21 | 3.69E-20 |
| TRAF2 | 1.457284832 | 8.680303868 | 3.84E-21 | 8.87E-20 |
| GBP2 | 1.621263466 | 9.407997112 | 7.47E-20 | 1.55E-18 |
| TNFRSF4 | 2.177454422 | 7.439858018 | 2.98E-19 | 5.28E-18 |
| NFIL3 | -1.135993181 | 10.17384653 | 3.05E-19 | 5.28E-18 |
| TAZ | 1.298176643 | 8.339840289 | 9.10E-19 | 1.46E-17 |
| LIG1 | 1.429479517 | 8.174357453 | 5.15E-17 | 7.65E-16 |
| CCR1 | -1.156439661 | 7.387792871 | 2.55E-16 | 3.53E-15 |
| CCL25 | 6.644685377 | 10.3444837 | 2.77E-15 | 3.60E-14 |
| TLR4 | -1.128606676 | 7.286378851 | 2.92E-14 | 3.37E-13 |
| SKAP1 | -1.322871034 | 7.909345575 | 1.22E-13 | 1.34E-12 |
| CKLF | 1.257737515 | 8.327964294 | 2.01E-13 | 2.09E-12 |
| CCL4 | -1.108231083 | 7.587337247 | 5.93E-13 | 5.88E-12 |
| MBL2 | -1.553606674 | 10.45586899 | 9.78E-13 | 9.25E-12 |
| CD1D | -1.304715237 | 7.799181007 | 1.23E-12 | 1.12E-11 |
| CD24 | 2.380467262 | 10.87695797 | 4.02E-12 | 3.22E-11 |
| TREM2 | 1.688791861 | 8.134079116 | 7.33E-12 | 5.64E-11 |
| JAG2 | 1.761600792 | 7.393027191 | 1.17E-11 | 8.70E-11 |
| RPS19 | 1.167386443 | 13.1096856 | 1.58E-11 | 1.10E-10 |
| MMP9 | 2.236323635 | 8.646996805 | 7.59E-11 | 5.09E-10 |
| RSAD2 | -1.321111412 | 7.216046709 | 1.34E-09 | 7.72E-09 |
| CCL2 | -1.120213504 | 8.735904922 | 2.64E-09 | 1.45E-08 |
| SEMA7A | 1.406230782 | 7.529908716 | 1.60E-08 | 7.91E-08 |
| SAA1 | -1.86254827 | 16.27338767 | 4.10E-08 | 1.94E-07 |
| CALCA | 3.732318659 | 7.759995575 | 6.36E-08 | 2.94E-07 |
| INHA | 3.373408812 | 7.237162114 | 1.51E-07 | 6.29E-07 |
| IKBKG | 1.109800137 | 7.742311083 | 2.14E-07 | 8.71E-07 |
| IL32 | 1.090090199 | 12.46711417 | 4.54E-07 | 1.72E-06 |
| RAB3D | 1.670433889 | 7.28503793 | 5.03E-07 | 1.87E-06 |
| PTGDR2 | 1.90730332 | 7.104301069 | 5.24E-07 | 1.89E-06 |
| APOA1 | -1.088294756 | 17.99597228 | 6.56E-07 | 2.31E-06 |
| CCL20 | 1.654619121 | 10.32507226 | 2.71E-06 | 8.67E-06 |
| TCF7 | 1.13300379 | 7.459322778 | 1.35E-05 | 4.00E-05 |
| CHST4 | -1.463884039 | 7.306907908 | 3.34E-05 | 9.15E-05 |
| APOA4 | 2.763713489 | 13.52450219 | 4.32E-05 | 0.000113617 |
| CTSE | 2.370867984 | 7.215942879 | 0.000104351 | 0.000258393 |
| PDCD1 | 1.421371472 | 7.085434383 | 0.000109808 | 0.000268706 |
| CD7 | 1.214272191 | 8.013437644 | 0.000147347 | 0.000348275 |
