# Supplementary material for: Comprehensive analysis of the MIR4435-2HG/miR-1-3p/MMP9/miR-29-3p/DUXAP8 ceRNA network axis in hepatocellular carcinoma
Source: Discov Oncol. 2021 Oct 7;12:38. doi: 10.1007/s12672-021-00436-3 (PMC8777520; doi:10.1007/s12672-021-00436-3)
Supplement: Supplementary file 2 — Additional file 2: Supplementary Table 2: The list of upstream miRNAs of five immune-DEmRNAs for hepatocellular carcinoma (DOCX 20 KB) [file 12672_2021_436_MOESM2_ESM.docx]

**Supplementary Table 2**

The list of upstream miRNAs of five immune-DEmRNAs for hepatocellular carcinoma

| miRNA ID | Database | Tissue | Target gene | Experimental methods |
| --- | --- | --- | --- | --- |
| hsa-mir-21-5p | miRBase | Liver | MMP9 | qRT-PCR |
| hsa-mir-29b-3p | miRBase | Liver | MMP9 | Luciferase reporter assay//qRT-PCR |
| hsa-mir-132-3p | miRBase | Liver | MMP9 | Luciferase reporter assay |
| hsa-mir-124-3p | miRBase | Liver | MMP9 | Western Blot |
| hsa-mir-141-3p | miRBase | Liver | MMP9 | Western Blot |
| hsa-mir-145-5p | miRBase | Liver | MMP9 | Western Blot, qPCR, Luciferase Reporter Assay, ELISA |
| hsa-mir-195-5p | miRBase | Liver | MMP9 | qPCR |
| hsa-mir-200a-3p | miRBase | Liver | MMP9 | Western Blot |
| hsa-mir-223-3p | miRBase | Liver | MMP9 | Western Blot |
| hsa-mir-34a-5p | miRBase | Liver | MMP9 | qPCR |
| hsa-mir-494-3p | miRBase | Liver | MMP9 | Western Blot |
| hsa-mir-497-5p | miRBase | Liver | MMP9 | Western Blot |
| hsa-mir-1-3p | miRBase | Liver | MMP9 | RPF-Seq, RNA-Seq |
| hsa-mir-101-3p | miRBase | Liver | MMP9 | Microarrays |
| hsa-mir-152-3p | miRBase | Liver | APLN | HITS-CLIP |
| hsa-mir-33b-3p | miRBase | Liver | APLN | HITS-CLIP |
| hsa-let-7a-5p | miRBase | Liver | APLN | HITS-CLIP |
| hsa-mir-126-3p | miRBase | Liver | APLN | Microarrays |
| hsa-mir-21-5p | miRBase | Liver | CCL20 | ELISA//Immunofluorescence//Immunohistochemistry//In situ hybridization//Luciferase reporter assay//qRT-PCR |
| hsa-mir-145-5p | miRBase | Liver | CCL20 | Luciferase Reporter Assay |
| hsa-mir-124-3p | miRBase | Liver | CCL20 | RNA-Seq, Microarrays |
| hsa-mir-101-3p | miRBase | Liver | CCL20 | Microarrays |
| hsa-mir-129-2-3p | miRBase | Liver | CCL20 | Microarrays |
| hsa-mir-194-5p | miRBase | Liver | CCL20 | Microarrays |
| hsa-mir-34a-5p | miRBase | Liver | CCL20 | Microarrays |
| hsa-mir-34c-5p | miRBase | Liver | CCL20 | Microarrays |
| hsa-mir-429 | miRBase | Liver | CCL20 | Microarrays |
| hsa-mir-449a | miRBase | Liver | CCL20 | Microarrays |
| hsa-mir-26a-5p | miRBase | Liver | TNFRSF4 | Microarrays |
| hsa-mir-34a-5p | miRBase | Liver | TRAF2 | PCR array |
| hsa-mir-1-3p | miRBase | Liver | TRAF2 | RPF-Seq |
| hsa-mir-31-5p | miRBase | Liver | TRAF2 | Microarrays |
