# Supplementary material for: Comprehensive analysis of the MIR4435-2HG/miR-1-3p/MMP9/miR-29-3p/DUXAP8 ceRNA network axis in hepatocellular carcinoma
Source: Discov Oncol. 2021 Oct 7;12:38. doi: 10.1007/s12672-021-00436-3 (PMC8777520; doi:10.1007/s12672-021-00436-3)
Supplement: Supplementary file 3 — Additional file 3: Supplementary Table 3: The hazard ratio for death and p value of upstream miRNAs in HCC (DOCX 19 KB) [file 12672_2021_436_MOESM3_ESM.docx]

**Supplementary Table 3**

The hazard ratio for death and p value of upstream miRNAs in HCC

| miRNA | HR | p-value | FDR |
| --- | --- | --- | --- |
| hsa-mir-126-3p | 0.55 | 0.00069 | 0.006875 |
| hsa-mir-29b-3p | 0.465 | 0.00073 | 0.006875 |
| hsa-mir-132-3p | 1.79 | 0.00093 | 0.006875 |
| hsa-mir-34a-5p | 0.56 | 0.0011 | 0.006875 |
| hsa-mir-194-5p | 1.57 | 0.0018 | 0.008333 |
| hsa-mir-101-3p | 0.58 | 0.002 | 0.008333 |
| hsa-mir-33b-3p | 1.67 | 0.0049 | 0.0155 |
| hsa-mir-21-5p | 1.65 | 0.0055 | 0.0155 |
| hsa-mir-141-3p | 1.65 | 0.0057 | 0.0155 |
| hsa-mir-429 | 1.63 | 0.0062 | 0.0155 |
| hsa-mir-1-3p | 0.6 | 0.0072 | 0.015577 |
| hsa-let-7a-5p | 0.51 | 0.0078 | 0.015577 |
| hsa-mir-449a | 0.54 | 0.0081 | 0.015577 |
| hsa-mir-195-5p | 0.64 | 0.011 | 0.018333 |
| hsa-mir-200a-3p | 1.57 | 0.011 | 0.018333 |
| hsa-mir-145-5p | 0.68 | 0.028 | 0.04375 |
| hsa-mir-497-5p | 0.68 | 0.047 | 0.068056 |
| hsa-mir-34c-5p | 1.42 | 0.049 | 0.068056 |
| hsa-mir-26a-5p | 1.37 | 0.092 | 0.121053 |
| hsa-mir-223-3p | 1.33 | 0.11 | 0.1375 |
| hsa-mir-124-3p | 0.73 | 0.19 | 0.22619 |
| hsa-mir-494-3p | 0.84 | 0.37 | 0.41 |
| hsa-mir-152-3p | 0.85 | 0.38 | 0.41 |
| hsa-mir-129-2-3p | 0.93 | 0.41 | 0.41 |
| hsa-mir-31-5p | 1.00E-04 | 0.41 | 0.41 |
